# Prognostic value of the AIP index in patients with severe aortic stenosis undergoing transcatheter aortic valve replacement

**DOI:** 10.3389/fnut.2026.1753594

**Published:** 2026-03-11

**Authors:** Xin-fan Lin, Ai-zhen Chen, Li-kang Ma, Si-ying Luo, Zhan-qiao Chen, Qi Chen, Tian-xin Lan, Qing-song Wu, Lin-feng Xie, Xing-feng Chen, Liang-wan Chen, Zhi-huang Qiu

**Affiliations:** 1 Department of Cardiovascular Surgery, Fujian Medical University Union Hospital, Fuzhou, Fujian, China; 2 Key Laboratory of Cardio-Thoracic Surgery (Fujian Medical University), Fujian Province University, Fuzhou, Fujian, China; 3 Fujian Provincial Center for Cardiovascular Medicine, Fuzhou, Fujian, China; 4 Department of Education Administration, Fujian Medical University Union Hospital, Fuzhou, Fujian, China

**Keywords:** all-cause mortality, aortic stenosis, atherogenic index of plasma, cardiovascular mortality, major adverse cardiovascular event, transcatheter aortic valve replacement

## Abstract

**Background:**

The atherogenic index of plasma (AIP) is a recognized predictor of cardiovascular risk, yet its prognostic relevance in patients with severe aortic stenosis (AS) undergoing transcatheter aortic valve replacement (TAVR) remains uncertain.

**Methods:**

This single-center retrospective study included 314 severe AS patients who underwent TAVR between 2019 and 2023. Participants were stratified into tertiles by preoperative AIP (Q1 < −0.12; Q2: −0.12 to 0.11; Q3 > 0.11). Outcomes included all-cause mortality, cardiovascular mortality, and major adverse cardiac and cerebrovascular events (MACCE). Multivariable Cox regression and restricted cubic spline (RCS) analyses assessed associations between AIP and clinical endpoints.

**Results:**

Over a median follow-up of 29 months (47 all-cause deaths, 34 cardiovascular deaths, and 67 MACCE events), Kaplan–Meier analysis demonstrated progressively poorer outcomes with increasing AIP tertiles (all log-rank *p* < 0.05). In multivariable Cox models, each 1-unit increase in AIP was independently associated with higher risks of all-cause mortality (aHR = 7.39, 95% CI 2.57–21.27), cardiovascular mortality (aHR = 11.24, 95% CI 3.25–38.90), and MACCE (aHR = 4.98, 95% CI 2.11–11.78). Restricted cubic spline analyses further confirmed significant linear dose–response relationships between AIP and all three endpoints (all P for nonlinearity > 0.05), with risk increasing progressively above reference levels around 0.44–0.45. Significant interactions were observed in current smokers and patients with coronary heart disease (P for interaction < 0.05), suggesting amplified AIP-associated risks in these subgroups.

**Conclusion:**

Elevated preoperative AIP is independently and linearly associated with increased mortality and MACCE risks in patients with severe AS undergoing TAVR. AIP may serve as a readily available metabolic biomarker providing supplementary prognostic information.

## Introduction

Aortic stenosis (AS), the most prevalent valvular pathology worldwide, exhibits a striking age-dependent epidemiology, with prevalence rates rising exponentially beyond 65 years of age. Current epidemiological data estimate 13.3 million global AS cases in 2021, with untreated severe AS portending particularly poor outcomes (1). While transcatheter aortic valve replacement (TAVR) has transformed severe AS management through its expanding clinical applications, critical gaps remain in our understanding of prognostic markers - especially those related to metabolic pathways (2–4).

The atherogenic index of plasma (AIP), calculated as log (TG/HDL-C), has emerged as a superior biomarker of cardiovascular risk, outperforming conventional lipid measures (TG, TC, LDL-C, HDL-C) in predicting atherogenic lipoprotein profiles (5, 6). Extensive clinical evidence positions AIP as a powerful predictor of cardiometabolic outcomes, demonstrating consistent associations with coronary artery disease, heart failure, stroke, and incident type 2 diabetes mellitus (7–9). The clinical utility of AIP derives from its unique capacity to quantify the atherogenic-to-protective lipoprotein ratio, a key determinant of vascular pathophysiology (10). Particularly relevant to AS, metabolic dysfunction has been directly implicated in valvular calcification and disease progression (11, 12).

Despite these well-established associations, the prognostic value of AIP in TAVR recipients remains undetermined—a critical knowledge gap given the expanding TAVR-eligible population. This study systematically evaluates the prognostic relevance of AIP in AS patients undergoing TAVR, with the objective of exploring its potential role as a metabolism-related biomarker that may provide supplementary information for preoperative outcome assessment in valvular heart disease management.

## Methods

### Study design and population

This single-center retrospective study analyzed consecutive patients undergoing TAVR procedures for severe AS at Fujian Medical University Union Hospital between January 2019 and December 2023. Severe AS was defined by: (1) aortic valve area ≤ 1.0 cm^2^, and (2) peak aortic jet velocity ≥ 4 m/s, or mean aortic valve gradient ≥ 40 mmHg. TAVR indication were determined by a multidisciplinary evaluation of age, estimated life expectancy, comorbidities, anatomical and procedural characteristics, feasibility of vascular access, the risks of operation, bioprosthetic valve durability, and the long-term outcomes. All procedures followed standard clinical guidelines, with prosthetic valve sizing determined by preoperative computerized tomography measurements and manufacturer specifications (2, 13).

**Figure 1:**
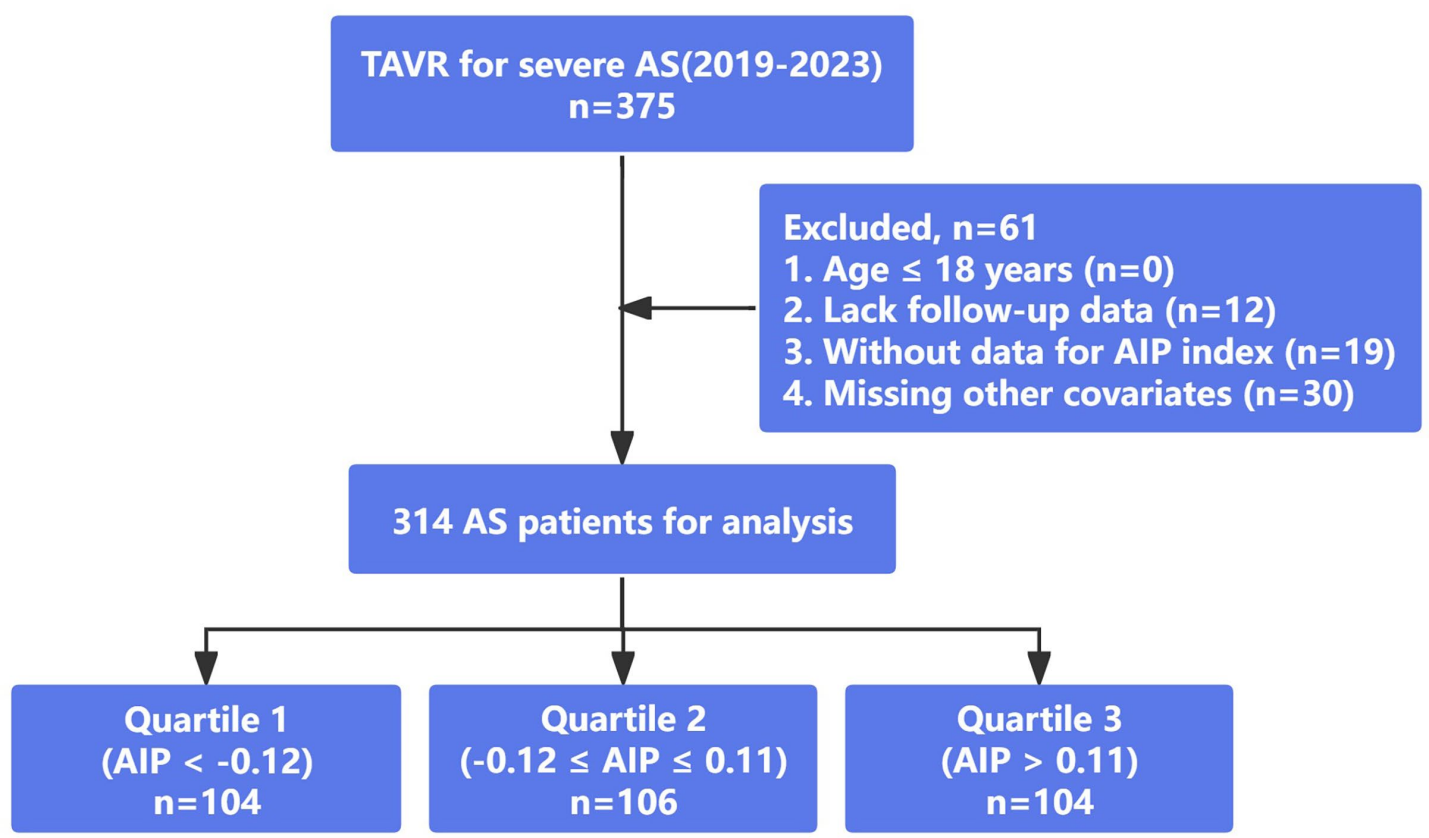
Flowchart of the study participants.

Of the 375 patients, 61 were excluded for meeting the exclusion criteria, i.e., (1) age ≤ 18 years (*n* = 0); (2) lack follow-up data (*n* = 12); (3) without data for AIP index (*n* = 19); (4) missing other covariates (*n* = 30). The final analysis included 314 patients stratified into preoperative atherogenic index of plasma (AIP) tertiles with the first tertile as reference: Quartile 1 (Q1, AIP < −0.12, *n* = 104), Quartile 2 (Q2, −0.12 ≤ AIP ≤ 0.11, *n* = 106), and Quartile 3 (Q3, AIP > 0.11, *n* = 104) (Figure 1). Baseline characteristics were generally comparable between included and excluded patients (Supplementary Table 1), suggesting no strong evidence of systematic selection bias. The study adhered to the Helsinki Declaration and received approval from the Medical Ethics Committees of Fujian Medical University Union Hospital. Due to the retrospective nature of the design, the requirement for informed consent was waived.

### Data collection and definitions

The demographic data, medical history, laboratory test data, echocardiography data, and procedural details of all patients were extracted from our electronic medical records. All comorbidities were defined based on ICD-10 codes according to medical diagnosis. All blood samples analyzed in this study were morning venous blood collected after a fast of at least 8 h. The AIP index was calculated using the formula: log_10_ (TG/HDL-C) (5).

### Endpoint and follow-up

The primary endpoints were all-cause mortality and cardiovascular mortality. The secondary endpoint was Major Adverse Cardiac and Cardiovascular Events (MACCE) including nonfatal stroke, nonfatal myocardial infarction, readmission for acute heart failure, and all-cause mortality during the follow-up (14). Patients were followed up for 5 years after discharge, with those who did not complete the full follow-up censored at the study’s end date.

### Statistical analyses

All data analyses were performed using R (version 4.3.2) and SPSS (version 27.0.1). A two-sided *p*-value<0.05 was considered statistically significant. Baseline characteristics were summarized using descriptive statistics: continuous variables with normal distribution were reported as mean ± standard deviation (SD), non-normally distributed variables as median (interquartile range, IQR), and categorical variables as counts (percentages). Intergroup comparisons were performed using one-way ANOVA for normally distributed continuous variables, the Kruskal-Wallis test for skewed data, and chi-square or Fisher’s exact tests for categorical variables, as appropriate, to evaluate differences across AIP index groups.

Event-free survival across AIP tertiles was compared using Kaplan–Meier (KM) analysis with log-rank testing. Cox proportional hazards regression models were used to evaluate independent associations between AIP and endpoints, expressed as hazard ratios (HR) with 95% confidence intervals (95% CI). Three models were constructed: Model 1, unadjusted; Model 2, adjusted for age and sex; and Model 3 further adjusted for variables in model 2 plus.

BMI, diabetes, coronary heart disease, and aortic valve gradient. Based on Model 3, restricted cubic spline (RCS) regression with 3 knots was applied to examine potential nonlinear relationships.

Subgroup analyses based on sex, age (≤ / > 70 years), BMI (≤ / > 24 kg/m^2^), left ventricular ejection fraction (LVEF) (< / ≥ 60%), current smoking status, hypertension, diabetes, and coronary heart disease were performed to investigate the consistency of the prognostic impact of AIP index on outcomes. Likelihood ratio tests were executed to examine modifications and interactions between subgroups. To strengthen the robustness of our findings, we conducted two complementary sensitivity analyses: replication of the primary analysis after excluding patients with 30-day mortality, and exclusion of patients with concurrent cancer or chronic kidney disease.

## Results

### Baseline characteristics

The baseline characteristics and procedure information of 314 patients with AS, stratified by AIP tertiles (Q1: AIP < −0.12, *n* = 104; Q2: −0.12 ≤ AIP ≤ 0.11, *n* = 106; Q3: AIP > 0.11, *n* = 104) are presented in Table 1. The cohort had a mean age of 70.91 ± 6.65 years, with 63.69% males. Patients in the highest AIP tertile (Q3) had significantly higher BMI (23.6 vs. 21.2 kg/m^2^, *p* < 0.001), and a higher prevalence of current smoking (23.1% vs. 9.6%, *p* = 0.025), diabetes (23.1% vs. 8.7%, *p* = 0.016), and coronary heart disease (9.6% vs. 1.9%, *p* = 0.046). Additionally, the use of lipid-lowering therapy was more common in the Q3 group. Echocardiography showed that the Q3 group exhibited more severe aortic stenosis, with a significantly elevated peak aortic jet velocity (4.70 ± 0.90 m/s, *p* < 0.001) and mean aortic valve gradient (56.7 ± 20.1 mmHg, *p* = 0.014). Regarding procedural complications, the incidence of new-onset atrial fibrillation increased significantly with higher AIP levels (*p* = 0.034).

**Table 1:** Baseline characteristic.

Variables	Overall *n* = 314	Q1 (AIP < −0.12, *n* = 104)	Q2 (−0.12 ≤ AIP ≤ 0.11, *n* = 106)	Q3 (AIP > 0.11, *n* = 104)	*p*-value
AIP index	−0.013 [−0.166, 0.197]	−0.233 [−0.310, −0.167]	−0.013 [−0.064, 0.050]	0.296 [0.206, 0.525]	<0.001
Demographic					
Age (years)	70.911 (6.650)	70.98 1(7.429)	71.104 (6.342)	70.644 (6.171)	0.875
Male, %	200 (63.694%)	63 (60.577%)	65 (61.321%)	72 (69.231%)	0.355
BMI, kg/m^2^	22.586 [20.679, 25.225]	21.168 [19.781, 23.804]	23.098 [21.591, 25.606]	23.600 [21.821, 25.675]	<0.001
NYHA class ≥ III, %	230 (73.248%)	71 (68.269%)	79 (74.528%)	80 (76.923%)	0.346
Current smoking status, %	49 (15.695%)	10 (9.615%)	15 (14.151%)	24 (23.077%)	0.025
Medical history					
Hypertension, %	163 (51.911%)	50 (48.077%)	58 (54.717%)	55 (52.885%)	0.611
Diabetes, %	49 (15.605%)	9 (8.654%)	16 (15.094%)	24 (23.077%)	0.016
Coronary heart disease, %	17 (5.414%)	2 (1.923%)	5 (4.717%)	10 (9.615%)	0.046
Atrial fibrillation, %	27 (8.599%)	8 (7.692%)	11 (10.377%)	8 (7.692%)	0.725
Chronic lung disease, %	50 (15.924%)	12 (11.538%)	17 (16.038%)	21 (20.192%)	0.233
Cerebral vascular disease, %	9 (2.866%)	2 (1.923%)	3 (2.830%)	4 (3.846%)	0.708
Chronic kidney disease, %	37 (11.783%)	8 (7.692%)	14 (13.208%)	15 (14.423%)	0.275
Laboratory test					
FPG (mmol/L)	4.820 [4.315, 5.538]	4.635 [4.130, 5.088]	4.800 [4.348, 5.315]	5.310 [4.553, 6.888]	<0.001
TC (mmol/L)	4.100 [3.288, 4.912]	4.040 [3.325, 4.613]	4.255 [3.430, 5.090]	4.060 [3.195, 4.897]	0.554
TG (mmol/L)	1.120 [0.820, 1.540]	0.770 [0.670, 0.912]	1.120 [0.903, 1.357]	1.860 [1.440, 2.780]	<0.001
LDL-C (mmol/L)	2.560 [2.012, 3.240]	2.450 [2.008, 3.020]	2.770 [2.082, 3.372]	2.510 [1.968, 3.337]	0.190
HDL-C (mmol/L)	1.140 [0.930, 1.390]	1.385 [1.230, 1.612]	1.125 [0.950, 1.322]	0.915 [0.770, 1.065]	<0.001
Albumin (g/L)	37.350 [34.800, 40.300]	37.150 [35.175, 40.425]	37.800 [34.875, 40.675]	37.300 [34.700, 39.600]	0.798
Serum creatinine (umol/L)	75.500 [65.000, 91.000]	74.000 [62.000, 86.250]	75.000 [65.000, 91.000]	78.500 [66.500, 98.000]	0.120
Echocardiography data					
Bicuspid aortic valve, %	38 (12.102%)	11 (10.577%)	15 (14.151%)	12 (11.538%)	0.713
LVPW (mm)	11.600 [10.300, 12.900]	11.200 [10.200, 12.600]	11.650 [10.300, 12.975]	11.750 [10.400, 12.725]	0.621
IVS (mm)	12.500 [11.300, 14.000]	12.400 [11.475, 14.000]	12.500 [11.100, 14.000]	12.550 [11.425, 14.000]	0.971
Peak aortic jet velocity (m/s)	4.500 (0.800)	4.300 (0.700)	4.600 (0.800)	4.700 (0.900)	<0.001
Aortic valve gradient (mmHg)	54.700 (20.300)	52.100 (19.800)	55.300 (20.500)	56.700 (20.100)	0.014
Moderate-to-severe AR, %	100 (31.847%)	30 (28.846%)	33 (31.132%)	37 (35.577%)	0.570
LVEF, %	61.900 [54.350, 67.500]	63.700 [52.500, 68.800]	61.650 [50.900, 67.500]	61.300 [55.400, 67.225]	0.522
Medication					
Lipid-lowering therapy, %	127 (40.446%)	33 (31.731%)	38 (35.849%)	56 (53.846%)	0.003
Statins	95 (30.255%)	25 (24.038%)	28 (26.415%)	42 (39.623%)	0.021
Ezetimibe	43 (13.694%)	11 (10.577%)	17 (16.038%)	15 (14.151%)	0.498
PCSK9 inhibitors	16 (5.096%)	3 (2.885%)	6 (5.660%)	7 (6.731%)	0.428
Anti-hypertension therapy, %	152 (48.408%)	47 (45.192%)	54 (50.943%)	51 (49.038%)	0.698
Anti-platelet therapy, %	20 (6.369%)	3 (2.885%)	6 (5.660%)	11 (10.577%)	0.071
Procedural details					
Bioprosthetic heart valve, %					0.620
Self-expanding valve	257 (81.847%)	82 (78.846%)	88 (83.019%)	87 (80.769%)	
Balloon-expandable valve	57 (18.152%)	22 (21.154%)	18 (16.981%)	17 (16.346%)	
Access, %					0.697
Transfemoral	258 (82.166%)	88 (84.615%)	85 (80.189%)	85 (81.731%)	
Other access	56 (17.834%)	16 (15.385%)	21 (19.811%)	19 (18.269%)	
Access approach, %					0.916
Puncture	301 (95.860%)	99 (95.192%)	102 (96.226%)	100 (96.154%)	
Cut-down	13 (4.140%)	5 (4.808%)	4 (3.774%)	4 (3.846%)	
Concomitant PCI, %	8 (2.548%)	1 (0.962%)	2 (1.887%)	5 (4.808%)	0.185
Paravalvular leak, %	2 (0.637%)	0	1 (0.934%)	1 (0.962%)	0.607
Permanent pacemaker, %	34(10.828%)	8 (7.692%)	11 (10.377%)	15 (14.423%)	0.290
Stroke, %	4 (1.274%)	1 (0.962%)	1 (0.934%)	2 (1.923%)	0.771
Myocardial infarction, %	1 (0.318%)	0	0	1 (0.962%)	0.363
Conversion to surgery, %	1 (0.318%)	0	1 (0.934%)	0	0.374
New atrial fibrillation, %	15 (4.777%)	1 (0.962%)	5	9 (8.654%)	0.034
Vascular complication, %	7 (2.229%)	1 (0.962%)	3 (2.830%)	3 (2.885%)	0.563

Values are represented as the median (interquartile range), means ± standard deviation or *n* (percentages).

AIP, atherogenic index of plasma; FPG, fasting plasma glucose; HDL-C, high-density lipoprotein cholesterol; IVS, interventricular septum; LDL-C, low-density lipoprotein cholesterol; LVEF, left ventricular ejection fraction; LVPW, left ventricular posterior wall; NYHA, New York heart association; PCI, percutaneous coronary intervention; TC, total cholesterol; TG, triglycerides.

### Association between AIP and endpoint events

**Figure 2:**
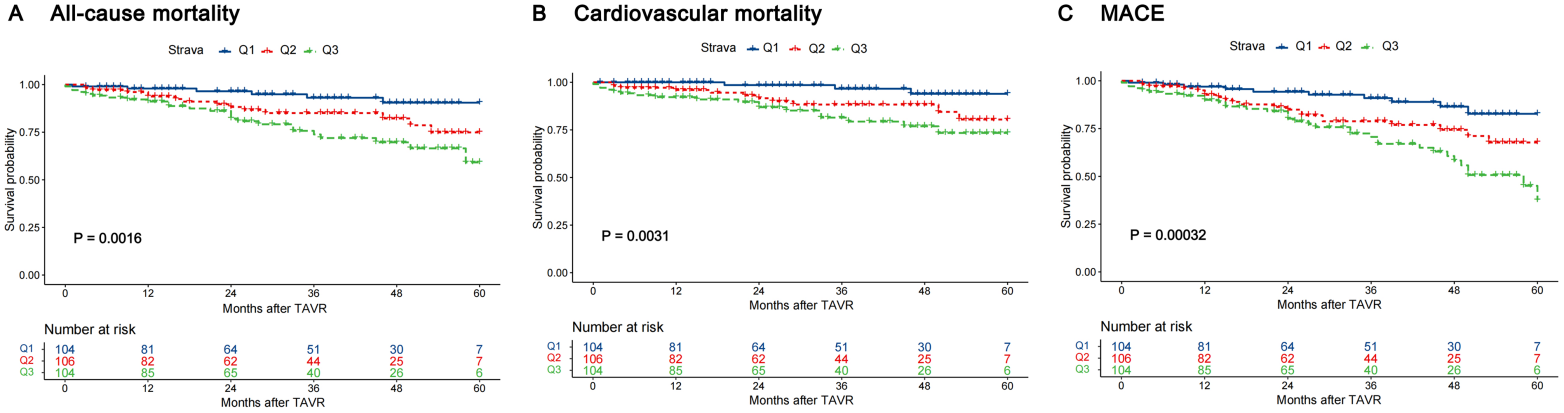
Kaplan–Meier curves for the AIP index tertiles. **(A)** All-cause mortality. **(B)** Cardiovascular mortality. **(C)** MACCE. AIP, atherogenic index of plasma; MACCE, major adverse cardiac and cardiovascular event.

During a median follow-up of 29.00 months (IQR: 13.00–48.00), the cohort experienced 47 (14.97%) all-cause mortality, 34 (10.83%) cardiovascular mortality, and 67 (21.34%) MACCE. Kaplan–Meier analysis showed progressively worse outcomes with higher AIP tertiles for all endpoints (log-rank *p* < 0.05, Figure 2).

Table 2 demonstrates that preoperative AIP levels were significantly associated with adverse outcomes in severe AS patients undergoing TAVR. After full adjustment, compared to Q1, Q2 and Q3 showed progressively higher risks of all-cause mortality (Q2 aHR = 3.29, 95%CI 1.05–10.30; Q3 aHR = 4.31, 1.43–13.00), cardiovascular mortality (Q2 aHR = 5.25, 1.14–24.23; Q3 aHR = 6.62, 1.47–29.94), and MACCE (Q2 aHR = 2.23, 1.08–3.52; Q3 aHR = 3.33, 1.49–7.45). When analyzed continuously, each 1-unit AIP increase corresponded to 639% (aHR = 7.39, 2.57–21.27), 1,024% (aHR = 11.24, 3.25–38.90), and 398% (aHR = 4.98, 2.11–11.78) elevated risks for all-cause mortality, cardiovascular mortality and MACCE respectively, demonstrating strong dose–response relationships.

**Table 2:** Cox regression analysis of the AIP index with all-cause, cardiovascular mortality and MACCE.

Outcomes exposure	Model 1		Model 2		Model 3	
	HR (95% CI)	*p*-value	HR (95% CI)	*p*-value	HR (95% CI)	*p*-value
All-cause mortality						
AIP (as continuous variable)	7.129 (2.963–17.150)	<0.001	7.490 (3.098–18.110)	<0.001	7.388 (2.566–21.273)	<0.001
AIP (as categorical variable)						
Q1	Reference		Reference		Reference	
Q2	2.901 (1.143–7.361)	0.025	2.718 (1.069–6.912)	0.036	3.294 (1.054–10.298)	0.040
Q3	4.156 (1.698–10.170)	0.002	4.054 (1.651–9.957)	0.002	4.309 (1.428–13.001)	0.010
Cardiovascular mortality						
AIP (as continuous variable)	10.130 (3.659–28.046)	<0.001	10.508 (3.798–29.067)	<0.001	11.240 (3.247–38.901)	<0.001
AIP (as categorical variable)						
Q1	Reference		Reference		Reference	
Q2	4.447 (1.267–15.611)	0.020	4.187 (1.191–14.726)	0.026	5.252 (1.138–24.234)	0.034
Q3	6.292 (1.853–21.368)	0.003	6.147 (1.805–20.931)	0.004	6.623 (1.465–29.943)	0.014
MACE						
AIP (as continuous variable)	5.013 (2.384–10.540)	<0.001	5.236 (2.479–11.059)	<0.001	4.983 (2.108–11.784)	<0.001
AIP (as categorical variable)						
Q1	Reference		Reference		Reference	
Q2	2.383 (1.134–5.008)	0.022	2.291 (1.087–4.828)	0.029	2.234 (1.077–3.517)	0.028
Q3	3.571 (1.764–7.231)	<0.001	3.568 (1.755–7.254)	<0.001	3.327 (1.485–7.453)	0.003

Model 1 was unadjusted.

Model 2 adjusted by age and sex.

Model 3 adjusted by model 2 + BMI, diabetes, coronary heart disease and aortic valve gradient.

AIP, atherogenic index of plasma; HR, hazard ratio; LVEF, left ventricular ejection fraction; MACCE, major adverse cardiac and cerebrovascular events; 95% CI, 95% confidence interval.

**Figure 3:**
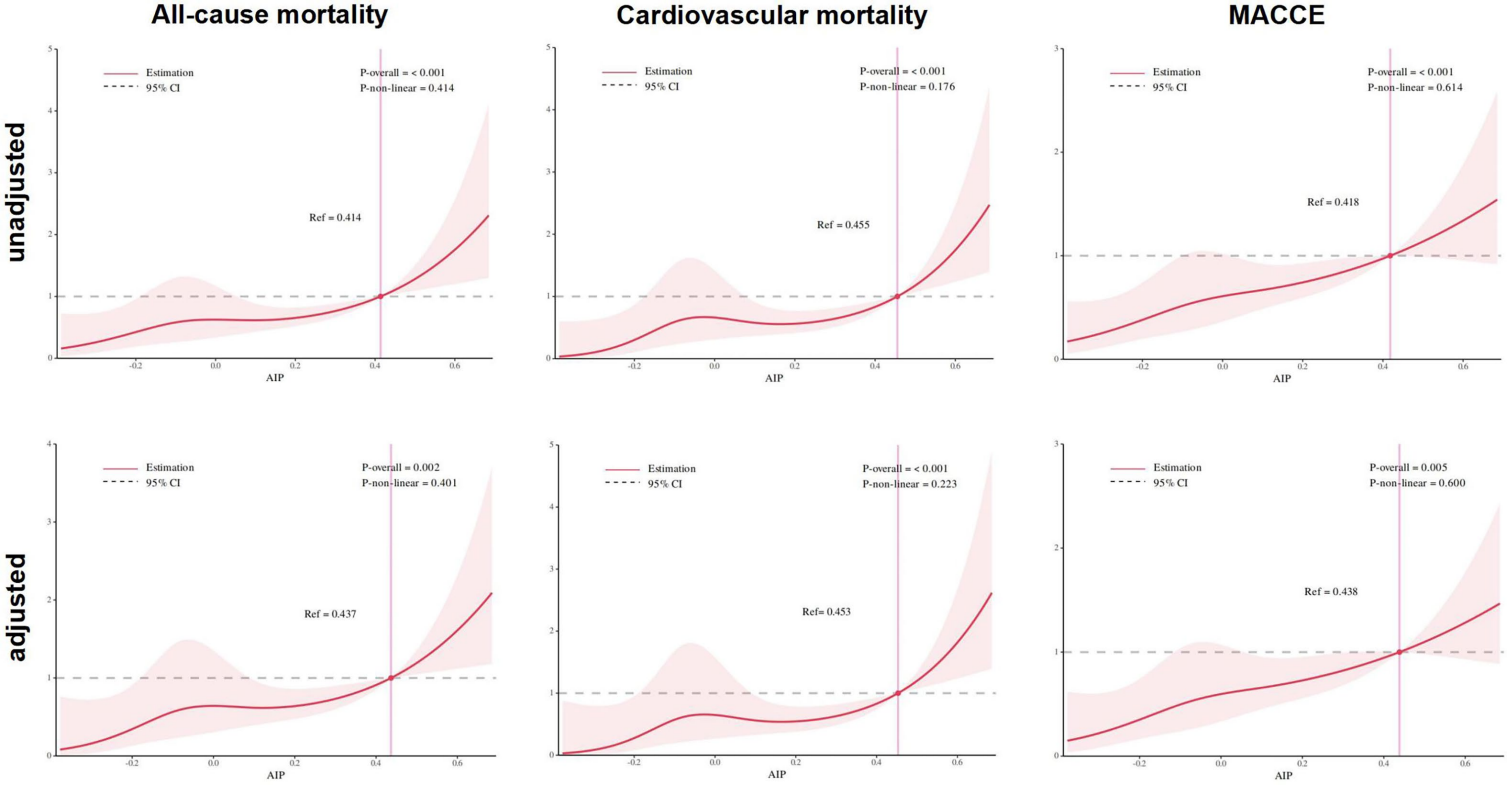
Restricted spline curves of the AIP index hazard ratios for the endpoints. **(A)** All-cause mortality unadjusted. **(B)** Cardiovascular mortality unadjusted. **(C)** MACCE unadjusted. **(D)** All-cause mortality adjusted. **(E)** Cardiovascular mortality adjusted. **(F)** MACCE adjusted. AIP, atherogenic index of plasma; CI, confidence interval; HR, hazard ratio; MACCE, major adverse cardiac and cardiovascular event; Ref, reference.

Both unadjusted and fully adjusted restricted cubic spline analyses demonstrated a significant positive association between increasing preoperative AIP levels and the risks of all-cause mortality, cardiovascular mortality, and MACCE after TAVR (all P-overall ≤ 0.005; Figure 3). Importantly, no evidence of a nonlinear relationship was observed for any endpoint (all P for nonlinearity > 0.05), supporting an approximately linear dose–response pattern across the range of AIP values. In the adjusted models, the hazard ratios began to rise progressively when AIP exceeded the reference levels (Ref ≈ 0.44 for all-cause mortality, Ref ≈ 0.45 for cardiovascular mortality, and Ref ≈ 0.44 for MACCE), suggesting that higher AIP values are associated with steadily increasing adverse outcome risk.

### Subgroup analyses

**Figure 4:**
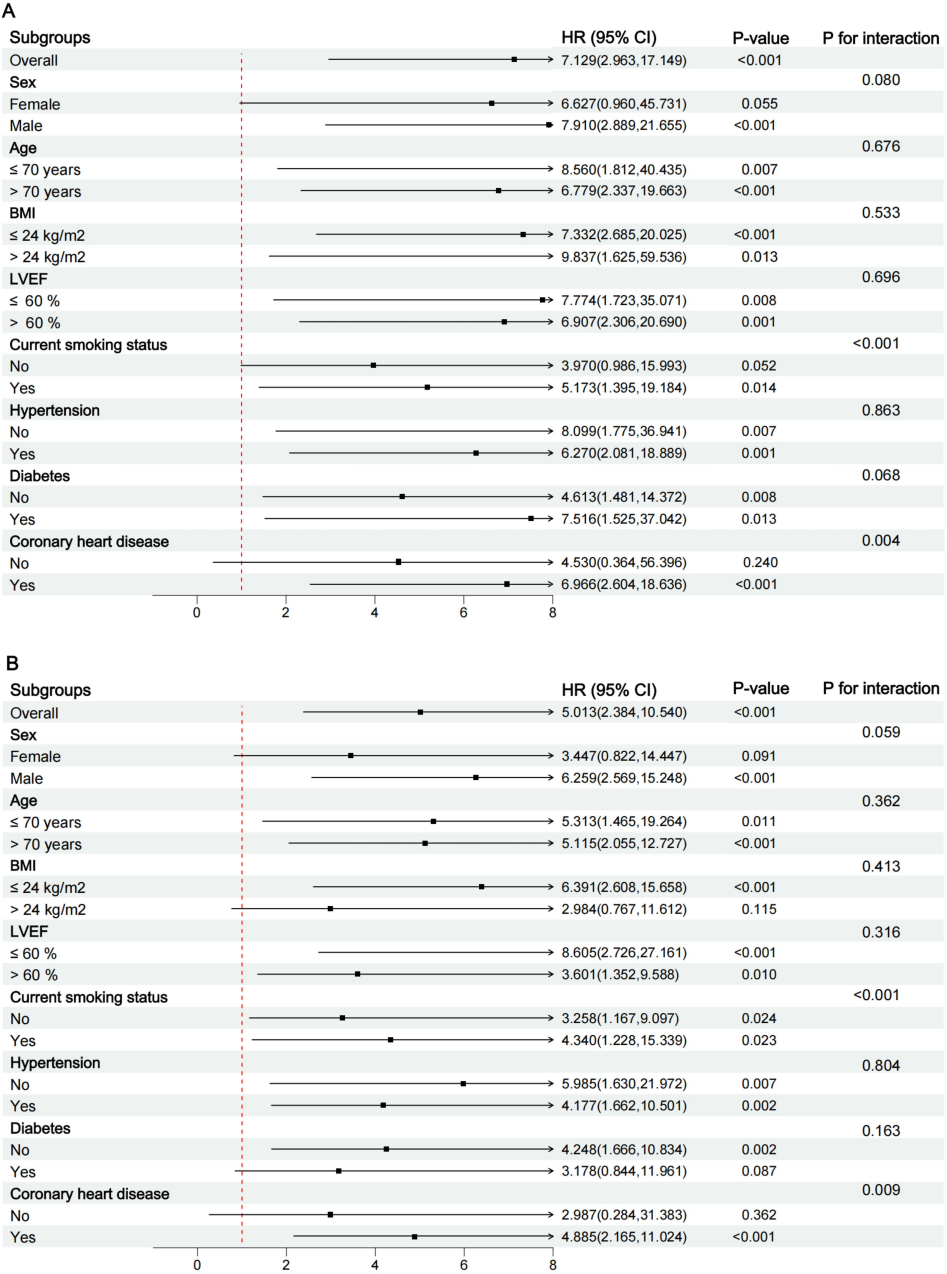
Subgroup analyses of the association between AIP index and outcomes. **(A)** All-cause mortality and **(B)** MACCE. MACCE, major adverse cardiac and cardiovascular event.

Subgroup analysis (Figure 4) showed that elevated preoperative AIP was consistently associated with increased all-cause mortality and MACCE across most strata. Notably, the association appeared markedly stronger in patients with pre-existing coronary heart disease (CHD) compared to those without. For all-cause mortality, the hazard ratio was significant in patients with CHD (HR = 6.97, 95%CI 2.60–18.64, *p* < 0.001) but was non-significant and imprecise in those without CHD (HR = 4.53, 95%CI 0.36–56.40, *p* = 0.240). A similar pattern was observed for MACCE, with a significant association in the CHD subgroup (HR = 4.89, 95%CI 2.17–11.02, *p* < 0.001) versus a non-significant one in the non-CHD subgroup (HR = 2.99, 95%CI 0.28–31.38, *p* = 0.362). This striking contrast suggests that pre-existing CHD may be a potential effect modifier of the relationship between AIP and postoperative prognosis. A significant interaction was, however, confirmed for current smoking status (P for interaction < 0.05 for both endpoints).

### Sensitivity analyses

Sensitivity analysis further confirmed the robustness of the results. After excluding patients who died within 30 days post-TAVR, significant differences persisted across all three AIP groups (all *p*-values<0.05, Supplementary Figure 1). Furthermore, the associations between AIP tertiles and clinical endpoints remained consistent with the primary findings after excluding patients with cancer or severe renal dysfunction (Supplementary Figure 2).

## Discussion

Our study provides evidence that elevated preoperative AIP levels are significantly associated with adverse clinical outcomes in patients with severe AS undergoing TAVR. The key findings are as follows: (1) Independent prognostic value—AIP consistently predicted all-cause mortality, cardiovascular mortality, and MACCE, and these associations remained significant after multivariable adjustment; (2) Dose–response pattern—RCS analyses supported an approximately linear relationship, with risks increasing progressively at higher AIP levels; (3) Robustness of findings—the observed associations were generally consistent across subgroup analyses and were further supported by multiple sensitivity analyses. Collectively, these results suggest that AIP, as a simple and readily available lipid-derived metabolic index, may provide supplementary prognostic information for TAVR candidates.

The management of severe AS has been revolutionized by the introduction of TAVR, which is now a recommended treatment option for a broad spectrum of patients, as evidenced by current clinical guidelines (15, 16). Epidemiological data demonstrate a significant age-dependent increase in AS prevalence, reaching 9.8% in octogenarians (aged 80–89 years) (17). While the prognostic value of conventional risk factors—including physiological reserve parameters [e.g., frailty syndrome and nutritional status (18, 19)] and major comorbidities (e.g., diabetes mellitus and chronic kidney disease (20, 21))—is well established, emerging evidence highlights the unique advantages of serum metabolic biomarkers in prognostic assessment. These biomarkers not only provide objective and quantifiable measurements but are also readily accessible in clinical practice. Multiple studies have confirmed the predictive value of the triglyceride–glucose index (TyG), an effective indicator of insulin resistance, along with other glycemic regulation markers including the hemoglobin glycation index (HGI), stress hyperglycemia ratio (SHR), and glucose variability (GV) for post-TAVR outcomes (22–24). Another promising lipid parameter, the AIP, was first introduced by Dobiasova and Frohlich (25) and has since emerged as a significant predictor of atherosclerotic burden and cardiovascular risk (6, 26). The pathobiology of degenerative aortic valve disease mirrors atherogenesis (27, 28), with both conditions exhibiting endothelial dysfunction, lipid deposition, calcification, and ossification (29). Intriguingly, statins—first-line agents for atherosclerosis—also decelerate AS progression in clinical studies (30, 31). In our cohort, patients with higher AIP levels presented with a more adverse cardiometabolic phenotype at baseline, including higher BMI and a greater prevalence of diabetes mellitus and coronary heart disease, suggesting that AIP may capture a broader metabolic risk burden. Moreover, baseline AS severity was significantly associated with AIP, with progressively higher peak aortic jet velocity and mean aortic valve gradient across AIP tertiles. These findings indicate that AIP may reflect both systemic metabolic risk and more advanced valvular disease at presentation.

Our study demonstrated that a higher AIP index was associated with a worse medium-term prognosis for patients with severe AS, even after TAVR treatment. Notably, after adjusting for potential confounding factors, patients in the highest AIP tertile had a 3.15-fold higher risk of MACCE compared to those in the lowest tertile. These findings are consistent with previously reported outcomes observed in coronary artery disease patients undergoing percutaneous coronary intervention (32). Elevated AIP levels are typically associated with an atherogenic dyslipidemia profile and systemic metabolic inflammation. Importantly, although TAVR effectively relieves valvular obstruction, post-procedural mortality and MACCE are frequently driven by pre-existing myocardial vulnerability and residual cardiometabolic risk rather than the valve lesion alone. In this context, elevated AIP may reflect two complementary pathways. First, AIP-related lipid dysregulation may contribute to the progression of degenerative aortic valve disease through lipid infiltration, inflammatory activation, and calcification, leading to more advanced stenosis and prolonged pressure overload prior to intervention. Such chronic afterload exposure may promote adverse myocardial remodeling, fibrosis, and diastolic dysfunction that are only partially reversible after TAVR, thereby predisposing patients to subsequent heart failure (33). Second, AIP may serve as a surrogate marker of residual atherosclerotic burden, including coronary artery disease and microvascular dysfunction, which may persist after TAVR and contribute to ischemic events and longer-term adverse outcomes. Therefore, AIP may provide supplementary prognostic information by capturing both valvular disease–related myocardial vulnerability and residual atherosclerotic cardiovascular risk (34).

Moreover, the clinical relevance of AIP may extend beyond preoperative risk assessment. As a readily available metabolic indicator, AIP may help identify patients who remain at increased cardiometabolic risk after TAVR and who may benefit from closer post-procedural follow-up. Notably, patients in the highest AIP tertile had a higher prevalence of statin use at baseline; however, their AIP levels remained significantly elevated and were associated with worse clinical outcomes, suggesting a persistent residual dyslipidemia burden characterized by elevated triglycerides and reduced HDL-C. Therefore, AIP may capture metabolic risk not fully addressed by traditional LDL-C–oriented management and serve as a complementary marker of residual cardiovascular risk in patients undergoing TAVR. Nevertheless, direct evidence supporting AIP-guided interventions to improve post-TAVR outcomes remains limited, and prospective studies are warranted to validate its clinical utility.

This study has several limitations. First, as a single-center retrospective investigation, residual confounding cannot be fully excluded, and the generalizability of our findings may be limited. Second, the median follow-up of approximately 30 months may be insufficient to assess long-term bioprosthetic valve durability and late cardiovascular events. Third, the lack of post-procedural or serial AIP measurements prevented further evaluation of longitudinal changes in AIP and their relationship with clinical outcomes. In addition, we did not formally examine the incremental prognostic value of AIP beyond existing risk models; therefore, its added role in clinical risk assessment requires further validation. Finally, our cohort was relatively younger, and atherosclerotic disease may have contributed more substantially to mortality, potentially amplifying the observed association between AIP and prognosis. Thus, larger prospective studies with longer follow-up and more diverse populations are warranted to confirm these findings.

## Conclusion

In summary, our study demonstrates that elevated preoperative AIP levels are independently associated with higher risks of all-cause mortality, cardiovascular mortality, and MACCE in patients with severe AS undergoing TAVR. The observed association follows an approximately linear dose–response pattern, suggesting that increasing AIP reflects a progressively greater cardiometabolic risk burden beyond traditional clinical factors. As a simple and routinely available lipid-derived index, AIP may provide supplementary prognostic value for preprocedural risk assessment and postoperative management. Future large-scale prospective studies are needed to validate these findings and to determine whether AIP-guided strategies could improve outcomes after TAVR.

## Data Availability

The raw data supporting the conclusions of this article will be made available by the authors, without undue reservation.
