# Supplementary material for: Prognostic value of the AIP index in patients with severe aortic stenosis undergoing transcatheter aortic valve replacement
Source: Front Nutr. 2026 Mar 11;13:1753594. doi: 10.3389/fnut.2026.1753594 (PMC13013486; doi:10.3389/fnut.2026.1753594)
Supplement: Supplementary file 1 [file Supplementary_file_1.docx]

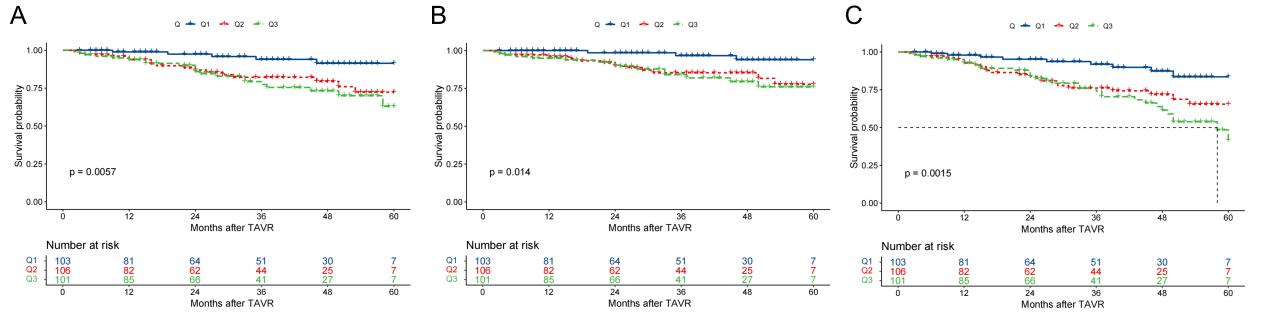


**Figure 1.** Kaplan-Meier Curves for AIP Index Tertiles After Excluding 30-Day Mortality


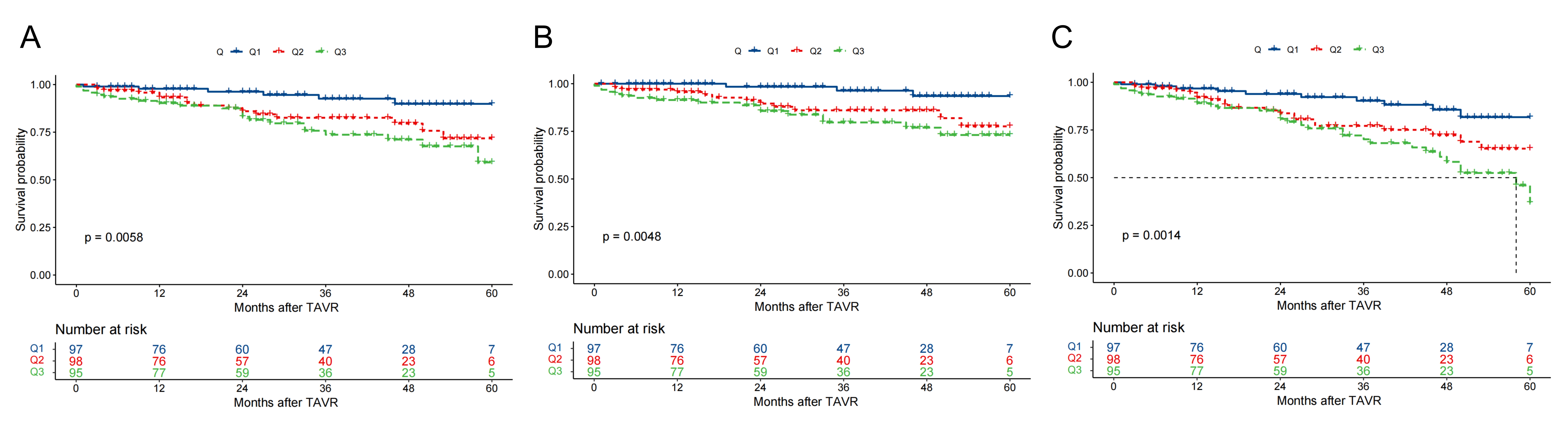


**Figure 2.** Kaplan-Meier Curves for AIP Index Tertiles After Excluding Patients with Cancer or Chronic Kidney Disease

**Table 1. Baseline characteristics of included and excluded patients**

| **Variables** | **Included(n=314)** | **Excluded(n=61)** | **P value** |
| --- | --- | --- | --- |
| Age | 70.911(6.650) | 71.655(7.410) | 0.433 |
| Sex | 200 (63.694%) | 40(65.574%) | 0.780 |
| BMI | 22.586 [20.679,25.225] | 23.531 [20.150,26.625] | 0.319 |
| Diabetes | 49 (15.605%) | 10(16.393%) | 0.636 |
| CHD | 17 (5.414%) | 2(3.279%) | 0.487 |
| CKD | 37 (11.783%) | 6(9.836%) | 0.662 |
| LVEF | 61.900 [54.350, 67.500] | 61.200 [49.650, 69.000] | 0.504 |
| Aortic valve gradient | 54.700 (20.300) | 54.300(18.500) | 0.887 |
